# Arthroscopic Bankart repair improves upper limb exercise capacity, shoulder function and quality of life

**DOI:** 10.55730/1300-0144.5361

**Published:** 2022-01-16

**Authors:** Figen DAĞ, Fehmi Volkan ÖZTUNA, Didem DERİCİ YILDIRIM, Özlem BÖLGEN ÇİMEN

**Affiliations:** 1 Department of Physical Medicine and Rehabilitation, Faculty of Medicine, Mersin University, Mersin, Turkey; 2 Department of Orthopedics and Traumatology, Faculty of Medicine, Mersin University, Mersin, Turkey; 3 Department of Biostatistic and Medical Informatics, Faculty of Medicine, Mersin University, Mersin, Turkey

**Keywords:** Bankart repair, exercise capacity, shoulder instability, quality of life

## Abstract

**Background/aim:**

Anterior glenohumeral instability is an important cause of shoulder disability. The aim of the present study was to investigate arm exercise capacity in patients with anterior glenohumeral instability before and after arthroscopic Bankart repair and to compare the results with those of healthy controls.

**Materials and methods:**

The patient group included a total of 11 males between the ages of 18 and 40 years. The control group consisted of 13 healthy males with an age range of 23 to 41 years. An incremental arm crank exercise test was performed to determine upper limb exercise capacity, as expressed by peak oxygen consumption (VO_2peak_). The shoulder function of the patients was evaluated by the Western Ontario Shoulder Instability Index (WOSI), and the quality of life was assessed with the Short Form-36 (SF-36). All evaluations were performed preoperatively, and at the postop 3^rd^ and 6^th^ months.

**Results:**

The patient group had lower VO_2peak_ and exhaustion duration at the preoperative assessment (p = 0.025 and p = 0.007, respectively). SF-36 domains were lower in patients (p < 0.05). There were significant differences in VO_2peak_ between preoperative and postop 6^th^-month measurements and between postop 3^rd^ and 6^th^-month measurements (p < 0.001 and p = 0.001, respectively). The total WOSI score increased from preoperative 50.27% to 57.77% at the postop 3^rd^ month, and to 65.56% at the final follow-up. Although improvements were detected in all SF-36 domains at postop follow-ups, they were not statistically significant except role limitations due to the physical problems domain (p = 0.006). There were no significant differences between controls and patients at the postop 3^rd^ and 6^th^ months with regard to exercise test parameters except the peak rating of perceived exertion.

**Conclusion:**

Shoulder function, exercise capacity, and quality of life were lower in the patient group and improved after arthroscopic Bankart repair. Clinicians should use the exercise capacity assessment for the evaluation of the recovery of shoulder function after providing stabilization.

## 1. Introduction

Anterior glenohumeral instability is a common clinical pathology in young and active individuals [1]. The fear of recurrent dislocation limits, the use of affected extremities may cause a decrease in shoulder function, level of physical activity, and quality of life in patients with glenohumeral instability [2–4]. Rehabilitation programs could help patients to maintain their physical activity level. However, the risk of recurrent dislocation, especially in young and active subjects, is around 90% [5]. Therefore, surgical stabilization may be considered for patients who failed to respond to conservative treatment or those at high risk for recurrent dislocation [3].

Determining shoulder functionality and physical capacity should be of considerable importance for clinicians. Performance-based evaluations may be essential in order to plan both physiotherapy programs [3]. Several researchers have investigated the upper-limb kinematics [6], muscle strength [3,5,7], range of motion (ROM) [3,4,7], and function of the shoulder [8] before and after the operation. However, to the best of our knowledge, no data are available concerning changes in arm exercise capacity in this specific patient group. Exercise capacity can be evaluated by measuring peak oxygen uptake (VO_2peak_) during an incremental arm crank ergometry test [9]. The determination of VO_2peak_ in patients with glenohumeral instability may provide an evaluation of pre and postop arm exercise capacity.

The purposes of the present study were to investigate upper-extremity exercise capacity, quality of life, and shoulder function in patients with anterior glenohumeral instability, to compare the results with healthy controls, and investigate the effects of arthroscopic Bankart repair. Our hypothesis is that arthroscopic Bankart repair capacity improves the quality of life and shoulder function, and normalizes upper-limb exercise capacity in 6-months.

## 2. Patients and methods

### 2.1. Subject

This prospective study included 18 nonsmoking male subjects with traumatic recurrent anterior shoulder instability. The inclusion criteria were: (1) being in an age range of 18–50 years; (2) having at least 3 recurrences of unilateral anterior shoulder dislocation; (3) having an isolated Bankart lesion. The exclusion criteria were: (1) presence of additional shoulder pathologies such as rotator cuff disorders, biceps tendon pathologies, Hill-Sachs lesion, glenohumeral ligament lesions; (2) having a history of previous revision surgery; (3) having other medical conditions that may affect the upper-limb exercise test (e.g., neurologic or movement disorders, chronic metabolic, neuromuscular or cardiopulmonary system diseases); (4) having a cognitive impairment that may prevent the individual from participating in exercise testing/rehabilitation program.

Of the 18 patients who met all the criteria, 3 were excluded because of missing exercise data and 4 were excluded because they did not continue the rehabilitation program, leaving a total of 11 patients available for analysis. The follow-up duration was 6-months for all patients.

The apprehension was graded with a visual analogue scale (VAS) (0–10), and the presence of avoidance was recorded during preoperative and postop follow-ups [10]. General joint laxity was evaluated with the Beighton Hypermobility Score [11]. Thirteen healthy age-matched nonsmoking male volunteers with no history of shoulder complaints or injuries were recruited as controls. Since the patient group consisted of only males, only male volunteers were included in the control group to eliminate gender-related differences. Ethical approval was obtained from the local ethics committee (2018–285) before the commencement of the study. Written informed consent was obtained from all patients.

### 2.2. Procedure

Shoulder function assessment: Each patient completed the shoulder instability questionnaire for the assessment of shoulder function. The patient’s experience related to shoulder instability during the latest week was evaluated using the WOSI, which was reported to be a valid, reliable, and disease-specific self-assessment tool developed for shoulder functions in patients with glenohumeral instability [12]. This index includes 21 items in 4 domains: physical symptoms, sport/recreation/work, lifestyle, and emotions. Each domain is specified on a scale of 0% to 100%, and 100% is the best score.

Quality of life: Quality of life was assessed via the Short Form-36 (SF-36) survey, which was indicated to be valid, reliable and consisted of 8 subdomains: Physical functioning, role limitations due to physical problems, pain, general health perception, vitality, social functioning, role limitations due to emotional problems, and mental health. Scores for each subdomain ranges from 0–100 points, where higher scores demonstrate better health levels [13].

Exercise capacity test: The participants were instructed not to take any food and caffeine, and smoke 4 h before the test and avoid strenuous exercises the day before the test. Each participant performed an incremental peak exercise test till exhaustion with an electronically braked arm ergometry (Monark 831 E; Varberg, Sweden) to determine exercise capacity (indicated by peak oxygen consumption; VO_2peak_). As shown in Figure, the participants were seated on a comfortable chair with back support and the center of the crank axis was in alignment with the acromion process to provide shoulder level at 90°.

The metabolic analyzer was calibrated before each test session with known gas concentrations (Quark-CPET, COSMED, Rome, Italy). The expired gases collected breath-by-breath during the exercise test. Heart rate (HR) was recorded during the test using a transmitter belt. The protocol began with a warm-up stage for 2-min by performing unloaded cranking. This period was followed by the first exercise stage that began with 30 W, and then increased by 10 W every minute until volitional exhaustion and they were unable to maintain the specified work rate. The subjects were instructed to keep the crank rate at 60 rev/min and verbally encouraged to maintain the test as long as possible. Finally, oxygen consumption (VO_2_), respiratory exchange ratio (RER), and HR data were averaged every 15 s to determine the peak values. The Borg’s scale was used to rate the perceived exertion [14]. All assessments were performed by the same physiotherapist, preoperatively and at the 3-month and 6-month follow-ups.

Arthroscopic Bankart repair: Arthroscopy was applied under general anesthesia using standard portals while the patient was in a beach chair position. All operations were performed by the same surgeon in accordance with basic principles. The glenoid neck was decorticated and debrided and the labrum was reinserted in its original position. The first suture bioabsorbable anchor was placed at the border of the glenoid at the 5 o’clock position. Additional anchors were placed at the glenoid edge at the 4 o’clock position, and if needed at the 3 o’clock position. Capsular plication was performed in patients with a history of eight or more episodes of dislocation.

Rehabilitation program: A standard postoperative rehabilitation program was applied by an experienced physiotherapist 3 times a week. The patients were asked to use a shoulder sling continuously for 3-weeks except during exercises. On the postop first day, active elbow, neck, and fingers Range of Motion (ROM) exercises were started with passive shoulder flexion, extension, and abduction. Pendulum exercises were started in the 3rd week together with forward active flexion (to 90°) and continued throughout weeks 3–6. During this period, external rotation was not allowed. Patients were also encouraged to perform isometric exercises for rotator cuff and deltoid during the 3–6 weeks. Full shoulder mobilization was allowed after 6-weeks. Between weeks 6–9, forward active flexion (130°), internal rotation (elevation in low-back), abduction (90°), external rotation (40°) were started.

### 2.3. Statistical analyses

The Shapiro-Wilk test was used to determine whether the variables were suitable for normal distribution. In cases where the distribution assumption is achieved, continuous variables are summarized as mean ± standard deviation, and continuous variables are presented as median (min–max) if the distribution assumption is not provided. The independent-samples t-test and Mann-Whitney U test were used according to the distribution assumption. Similarly, in the comparison of the two dependent groups, the paired samples t-test and Wilcoxon test were used depending on the distribution assumption. Repeated Measures of ANOVA were used to investigate differences between repeated measurements. Spearman correlation coefficient was calculated to determine the correlation between two variables. Analyses were performed with the Statistica v.13.3.1 program. Any p less than 0.05 were accepted as statistically significant.

## 3. Results

Baseline characteristics of the patients at the time of the operation are presented in Table 1. Both groups were similar in terms of age (p = 0.683), height (p = 0.554), body weight (p = 0.064), and body mass index (p = 0.083). The preoperative and postoperative levels of apprehension were significantly different (preoperative: 7.36 ± 2.38 vs. postop 3^rd^ month: 3.45 ± 2.84, p < 0.001 and preoperative: 7.36 ± 2.38 vs postop 6^th^ month: 2.50 ± 2.41, p < 0.001). Apprehension score decreased by 53% at the postop 3^rd^ and 66% at the postop 6^th^ month compared to the preoperative period. There was no significant difference between postop 3^rd^ and 6^th^-month values (p = 0.21).

Peak values of the exercise test for patients and controls at the preoperative assessment are presented in Table 2. Controls demonstrated significantly greater VO_2peak_ value than patients (p = 0.025). However, there was no significant difference between groups in terms of RER_peak_ (p = 0.643) and HR_peak_ (p = 0.094). Patients exhausted in a shorter time than controls (p = 0.007). Controls demonstrated significantly lower ratings of perceived exertion than the patient group (p = 0.008).

The preoperative exercise test results of the patients, at the postop 3^rd^ and 6^th^ months are demonstrated in Table 3. The preoperative mean VO_2peak_ (mL/min per kg) values for the patient group were 20.85 mL/min per kg. It improved from 1.51 mL/min per kg and 5.57 mL/min per kg to 22.36 mL/min per kg and 26.42 mL/min per kg at the postop 3^rd^ and 6^th^ months, respectively. There were significant differences in VO_2peak_ (mL/min per kg) value between preoperative measurement and the measurement at the postop 6^th^ month and between measurements at the postop 3^rd^ and 6^th^ month (p < 0.001 for both). Time to exhaustion was longer at the postop 6^th^ month than the preoperative measurement (p = 0.029).

There were no significant differences between controls and patients at the postop 3^rd^ and 6^th^ months with regard to exercise test parameters except the peak rating of perceived exertion (p > 0.05). It was significantly higher at the postop 3^rd^ and 6^th^ months compared to the preoperative measurement (p = 0.021 and p =0.040, respectively) (Table 4).

Significant improvements in sports/recreation/work and emotion domains of WOSI were noted between preoperative assessment and the postop 6^th^-month assessment (p = 0.036 and p = 0.005, respectively) and between postop 3^rd^ and 6^th^-month assessments (p = 0.012 and p = 0.021, respectively). The total WOSI score increased from 50.27% preoperatively to 57.77% at the postop 3^rd^ month and 65.56% at the final follow-up. However, these increments were not statistically significant (p = 0.068) (Table 3). There was no statistically significant correlation between the total WOSI score and VO_2peak_ (mL/min per kg) value during the follow-up period (p > 0.05).

All SF-36 subscales were statistically significantly lower in the patient group than controls (p < 0.05) (Table 2). Although improvements were detected for all SF-36 subscales at postop follows-up, they were not statistically significant except role limitations due to the physical problems domain (p = 0.006). Postop scores at the 3^rd^ and 6^th^ months were greater than the preop score (p = 0.032 for both) (Table 3).

## 4. Discussion

This study aimed to investigate the effects of arthroscopic Bankart repair on arm exercise capacity in patients with anterior glenohumeral instability. To the best of our knowledge, this is the first study that evaluated objectively the exercise capacity of patients with anterior glenohumeral instability with an exercise test before and after arthroscopic Bankart repair. The most important finding of the present study is that VO_2peak_ (mL/min per kg) increased 7.24% at the postop 3^rd^ month and 26.71% at the postop 6^th^ month compared to baseline after Bankart repair. The mechanism might be related to reestablished joint homeostasis. It has been reported that deterioration of the capsuloligamentous complex of the shoulder after dislocation has a harmful impact on proprioception, which may recover after glenohumeral joint repair [15]. The restoration of the capsuloligamentous balance might affect the muscles’ performance via normalized proprioception.

Arthroscopic Bankart repair with a suitable rehabilitation program could help patients to return to the previous physical activity level and increase their quality of life. Traditionally, the success of arthroscopic surgery is evaluated with isometric-isokinetic muscle strength or ROM. This research indicates that exercise capacity could be a new approach for evaluating the functional performance of upper-extremity, which is thought to be mediated by the proprioceptive input [15–17]. The present study demonstrated that exercise capacity was lower in patients with anterior glenohumeral instability compared with healthy controls at the preoperative assessment. Exercise capacity is the maximum amount of physical effort that an individual can maintain [18]. It is a reliable and valid indicator and used to detect the patients’ cardiovascular fitness for cardiopulmonary and musculoskeletal pathologies [19]. Instability patients avoid using the affected side in their daily life due to pain, fear of dislocation, and kinesiophobia (high fear of movement and reinjury) [2–5]. As a result of this avoidance, daily living activities and also upper-limb exercise capacity might be affected. Exercise capacity of patients improved at the postop 6^th^ month compared to the preoperative and postop 3^rd^-month assessments. VO_2peak_ values were nearly normal compared to healthy controls at the postop 3^rd^ and 6^th^ months. At preoperative and postop 3^rd^ month evaluations, patients’ exhaustion time was shorter than controls and approached gradually to controls at the 6^th^ month. After the test was terminated, the patients stated that they felt pain and disturbance at their shoulders. Therefore, we consider that especially shoulder pain and kinesiophobia during exercise test might be responsible for the shorter exhaustion time and this situation might be reflected in exercise capacity values in the patient group. Pain and kinesiophobia have been reported to be related to physical capacity [20,21]. Moreover, psychological variables such as stress level and kinesiophobia have also been associated with disability [22]. Kinesiophobia in musculoskeletal diseases has been highly studied [21–23]. Although there is a limited number of studies on shoulder instability [24], it is a common complaint in orthopedic and physical therapy clinics.

Patients who have undergone glenohumeral instability surgery usually return to the normal physical activity level in approximately 6-months [7]. According to the American Society of Shoulder and Elbow Therapists’ Consensus Rehabilitation Guideline, it is recommended that patients should gain the full range in all directions by the postop 12th week [25]. Buckwalter et al. reported that 76% of the patients have returned to baseline active range of motion (AROM) values and %98 of them returned to baseline muscle strength values at an average of postop 5.3-months [9]. Augustsson et al. [3] evaluated the AROM of 56 patients and found no significant difference on the unaffected shoulder compared with the affected side at post-op 6^th^-month assessment. Therefore, we performed the last follow-up in the 6^th^ month. Tahta et al. [4] found that there was no significant difference in AROM compared to the contralateral side 2 years after Bankart repair. Although AROM was not measured in the current study, no problems related to the limitation in the shoulder during the exercise test with arm crank ergometry were recorded in any period.

Generally, long-term results of muscle strength have been evaluated after surgery for instability cases [4,26,27]. Isokinetic muscle strengths only at internal and external rotations were found to be significantly lower when compared with the unaffected side [4,26,27]. Similarly, Meller et al. [28] reported decreased external rotation and abduction muscle strength after surgery at the 2-year follow-up. Considering the muscle strength values, when the affected side and the unaffected side are compared, it is seen that shoulder instability surgery has no significant effect on upper-extremity muscle strength [3]. Rhee et al. [9] have reported that muscle strength improved to the level of the contralateral side at the postop 6^th^ month. A minimal loss in ROM and muscle strength of external rotation and/or internal rotation does not prevent patients to return to their physical activities after postop 6-months [7]. Therefore, we did not evaluate muscle strength but most of our patients were young and physically active subjects, who participated either in sportive activity or high-demand works. It is suggested that physically active individuals might be able to compensate for strength insufficiency during functional activities [29]. In publications reporting muscle strength deficits, it has been stated that there were deficits only in rotational muscle strength [4,26,28]. We assumed that postop muscle strength did not affect our results, considering that there was no rotational movement during the arm crank ergometer.

It has been reported that shoulder instability might lead to disability in daily life activities [3,28,30]. Shoulder-specific questionnaires are administered differently from general health quality questionnaires, and it was suggested that general and disease-specific questionnaires should be used together [30]. The present study demonstrated that the quality of life of the patient group was worse than that of the healthy controls. Improvement was observed in SF-36 after arthroscopic repair. Especially improvement in the score of role limitation due to the physical problems subgroup was significant. We think that the decrease in role limitation due to physical problems might be reflected in the exercise capacity of the patients. We obtained better WOSI scores at postop 3^rd^ and 6^th^-month evaluations. Better WOSI scores indicate patients’ healing and significant clinical improvement at follow-up periods [3]. Although WOSI scores and exercise capacity improved independently in time, no correlation was found between WOSI score and VO_2peak_ for none of the follow-up periods, suggesting that subjective scores of shoulder function may not be related to exercise capacity in the patient group.

The present study has some limitations. First of all, this study was a prospective study with small sample size. It was needed to include as many patients as possible from both genders. But there are some studies that have been done with a similar sample size [6,28,31,32]. We considered AROM and muscle strength were not deterministic values for this patient group, further studies are needed to reveal the potential relationship between exercise capacity and these parameters in patients with glenohumeral instability.

The most important strength of this study was that it objectively measured the exercise capacity of the patients with anterior glenohumeral instability after arthroscopic Bankart repair. We, therefore, recommend the use of exercise capacity assessment for the evaluation of the recovery of shoulder function after providing stabilization. Further research studies are warranted to detect the factors that may affect upper-limb exercise capacity in patients with anterior glenohumeral instability. In conclusion, shoulder function, upper-limb exercise capacity, and quality of life decrease in patients with anterior glenohumeral instability, and they recover after successful Bankart repair.

## Figures and Tables

**Figure:**
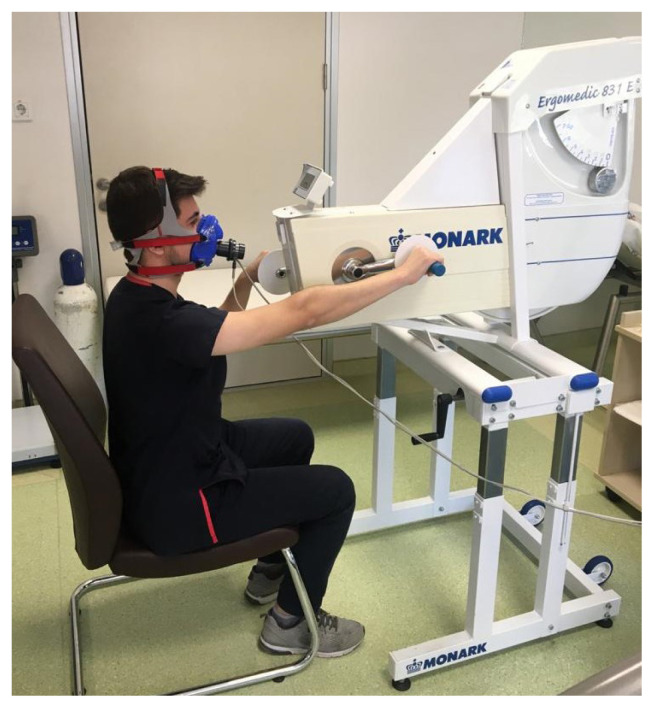
Exercise capacity assessment with arm crank ergometry.

**Table 1:** Baseline characteristics of the patients.

	No. of Patients (%)
Operative side	
Dominant	8 (73)
Nondominant	3 (27)
No. of instability episode	
3	4 (37)
3–8	5 (45)
>8	2 (18)
Ligament laxity	
Positive	1 (9)
Negative	10 (91)
Time to surgery	
≤3 mo	1 (9)
4–12 mo	5 (45.5)
>12 mo	5 (45.5)

**Table 2:** Exercise capacity and SF-36 variables for patients and controls at preoperative evaluation.

	Patients group (n = 11)	Controls (n = 13)	P
**Exercise capacity parameters**			
VO_2peak_ (mL/min per kg)	20.85 ± 3.20	24.97 ± 4.84	**0.025** *
VO_2peak_(mL/min)	1612.34 ± 187.19	2049.52 ± 476.32	**0.008** *
Peak respiratory exchange ratio	1.00 ± 0.09	1.02 ± 0.09	0.643
Peak heart rate	145.10 ± 19.45	157.83 ± 16.17	0.094
Time to exhaustion (min)	8.52 ± 1.03	11.48 ± 3.19	**0.007** *
Peak rating of perceived exertion	17.30 ± 1.26	15.58 ± 1.49	**0.008** *
**SF-36**			
Physical functioning	63.63 ± 20.98	96.15 ± 5.82	**<0.001**
Role limitations due to physical problems	11.36 ± 20.50 0.00 (0.00–50.00)	98.07 ± 6.93 100 (75.00–100.00)	**<0.001**
Pain	47.95 ± 36.92	89.42 ± 13.88	**0.004** *
Mental health	49.81 ± 21.19	74.76 ± 15.08	**0.003** *
Vitality	46.36 ± 25.69	70.00 ± 14.86	**0.016** *
Social functioning	63.63 ± 22.67	93.26 ± 9.70	**<0.001** *
Role limitations due to emotional problems	30.29 ± 37.87 33.30 (0.00–100)	94.86 ± 12.54 100.00 (66.50–100)	**<0.001**
General health perception	45.45 ± 19.80	74.23 ± 12.88	**<0.001** *

* p < 0.05. VO2peak; Peak oxygen consumption, SF-36: Short Form-36.

**Table 3:** Preoperative and postop 3^rd^ and 6^th^-month exercise capacity, WOSI, and SF-36 values.

	Preoperative	3^rd^monthpostop	6^th^monthpostop	P
Exercisecapacityparameters				
VO_2peak_(mL/minperkg)	20.85±3.20^b^	22.36±2.07^c^	26.42±2.28	**<0.001**
VO_2peak_(mL/min)	1612.34±187.19^b^	1704.24±312.79	1980.08±253.70	**0.002** *
Peakrespiratoryexchangeratio	1.00±0.09	1.07±0.06	1.03±0.06	0.100
Peakheartrate	145.10±19.45	154.20±20.71	160.90±19.03	0.148
Timetoexhaustion(min)	8.52±1.03^b^	9.46±2.40	10.45±2.03	**0.047** *
Peakratingofperceivedexertion	17.30±1.26	16.97±1.18	17.20±0.97	0.704
WOSI				
Physicalsymptoms	39.06±18.80	46.50±19.47	52.44±22.38	0.151
Sports/recreation/work	59.87±20.50^b^	53.69±23.18^c^	78.76±17.55	**0.023** *
Lifestyle	66.71±17.33	62.99±25.54	73.34±20.52	0.351
Emotions	53.11±23.65^b^	60.88±26.02^c^	81.27±14.63	**0.003** *
TotalWOSIscore	50.27±16.83	57.77±21.47	65.56±15.68	0.068
SF-36				
Physicalfunctioning	63.63±20.98	72.00±23.47	73.50±15.81	0.344
Rolelimitationsduetophysicalproblems	11.36±20.50^a^,^b^ 0.00(0.00–50.00)	50.00±41.83 50.00(0.00–100.00)	42.50±27.50 25.00(25.00–100.00)	**0.006** *
Pain	47.95±36.92	53.50±31.08	60.25±23.57	0.468
Generalhealthperception	45.45±19.80	56.50±15.50	57.00±15.68	0.128
Vitality	46.36±25.69	56.00±15.45	45.00±15.81	0.193
Socialfunctioning	63.63±22.67	73.75±21.97	68.75±17.89	0.282
Rolelimitationsduetoemotionalproblems	30.29±37.87 33.30(0.00–100.00)	53.29±40.02 33.30(0.00–100.00)	53.32±34.00 33.30(0.00–100.00)	0.084
Mentalhealth	49.81±21.19	60.40±18.62	53.20±18.68	0.096

* p<0.05. VO_2peak_; Peak oxygen consumption; WOSI: Western Ontario Shoulder Instability Index; SF-36: Short Form-36

a shows the difference between preoperative and postop 3^rd^-month assessments

b shows the difference between preoperative and postop 6^th^-month assessments

c shows the difference between postop 3^rd^ and 6^th^-month assessments

**Table 4:** Peak values of variables for healthy controls and patients at postop 3^rd^ and 6^th^ months (mean ± SD).

	Controls	Patients Postop 3^rd^ month	Patients Postop 6^th^ month
VO_2peak_ (mL/min per kg)	24.97 ± 4.84	22.36 ± 2.07	26.42 ± 2.28
VO_2peak_(mL/min)	2049.52 ± 476.32	1704.24 ± 312.79	1980.08 ± 253.70
Peak respiratory exchange ratio	1.02 ± 0.09	1.07 ± 0.06	1.03 ± 0.06
Peak heart rate	157.83 ± 16.17	154.20 ± 20.71	160.90 ± 19.03
Time to exhaustion (min)	11.48 ± 3.19	9.46 ± 2.40	10.45 ± 2.03
Peak rating of perceived exertion	15.58 ± 1.49*†	16.97 ± 1.18	17.20 ± 0.97

VO_2peak_; Peak oxygen consumption

* shows the difference between controls and patients at the postop 3^rd^ month

† shows the difference between controls and patients at the postop 6^th^ month
